# A systematic review and meta-analysis of randomized controlled trials for the reduction of surgical site infection in closed incision management versus standard of care dressings over closed vascular groin incisions

**DOI:** 10.1177/1708538119890960

**Published:** 2020-01-19

**Authors:** Alexander Gombert, Ellen Dillavou, Ralph D’Agostino, Leah Griffin, Julie M Robertson, Mark Eells

**Affiliations:** 1 Vascular Surgery, European Vascular Center Aachen-Maastricht, University Hospital RWTH Aachen, Aachen, Germany; 2 Vascular Surgery, Duke Regional Hospital, Duke University Medical Center, Durham, NC, USA; 3 Biostatistical Sciences, Wake Forest School of Medicine, Winston-Salem, NC, USA; 4 KCI, San Antonio, TX, USA

**Keywords:** Meta-analysis, vascular surgery, standard of care, negative pressure wound therapy

## Abstract

**Objective:**

Surgical site infection after groin incision is a common complication and a financial burden to patients and healthcare systems. Closed incision negative pressure therapy (ciNPT) has been associated with decreased surgical site infection rates in published literature. This meta-analysis examines the effect of ciNPT (PREVENA™ Incision Management System; KCI, San Antonio, TX) versus traditional postsurgical dressing use in reducing surgical site infection rates over closed groin incisions following vascular surgery.

**Methods:**

A systematic literature search using PubMed, OVID, EMBASE, and QUOSA was performed on 3 January 2019, by two independent researchers and focused on publications between 1 January 2005 and 31 December 2018. The review conformed to the statement and reporting check list of the Preferred Reporting Items for Systematic Reviews and Meta Analyses. Inclusion criteria included abstract or manuscript written in English, published studies, conference abstracts, randomized controlled trials (RCTs), ciNPT usage over closed groin incisions in vascular surgery, comparison of ciNPT use and traditional dressings, study endpoint/outcome of surgical site infection, and study population of >10. Characteristics of study participants, surgical procedure, type of dressing used, duration of treatment, incidence of surgical site infection, and length of follow-up were extracted. Weighted odds ratios and 95% confidence intervals were calculated to pool study and control groups in each publication for analysis. Treatment effects were combined using Mantel-Haenszel risk ratios, and the Chi-Square test was used to assess heterogeneity. Overall, high-risk patients, normal-risk patients, and Szilagyi I, II, III outcomes were assessed between ciNPT and control groups. The Cochrane Collaboration tool was utilized to assess the risk of bias for all studies included in the analysis.

**Results:**

A total of 615 articles were identified from the literature search. After removal of excluded studies and duplicates, six RCT studies were available for analysis. In these studies, a total of 362 patients received ciNPT, and 371 patients received traditional dressings (control). Surgical site infection events occurred in 41 ciNPT patients and 107 control patients. The heterogeneity test was nonsignificant (*p* > 0.05). The overall RCT meta-analysis showed a highly significant effect in favor of ciNPT (OR = 3.06, 95% CI [2.05, 4.58], *p* < 0.05). High-risk, normal-risk, Szilagyi I, and Szilagyi II meta-analyses were also statistically significant in favor of ciNPT use (*p* < 0.05). The varying RCT inclusion/exclusion criteria, such as differences in procedure types, and patient populations form the major limitations of this study.

**Conclusions:**

A statistically significant reduction in the incidence of surgical site infection was seen following ciNPT usage in patients undergoing vascular surgery with groin incisions.

## Introduction

Vascular surgical procedures, including lower extremity arterial surgery, involve standard access via a longitudinal groin incision, which may be frequently related to wound complications, lymphatic leakage, and surgical site infections (SSIs).^1^ SSI after groin incision is common and creates clinical complications and financial burden to patients and healthcare systems.^2^ With groin incisions, surgical site complications may result in limb loss and increased risk of death, with rates as high as 44%.^3^ According to the existent literature, adherence to SSI prevention measures can reduce their prevalence significantly.^4,5^

Recently, application of negative pressure therapy over clean, closed surgical incisions (closed incision negative pressure therapy, ciNPT) has been reported in various settings to be associated with a reduced rate of SSIs.^6–12^ However, these recent studies have been published in multiple surgical procedures and multiple ciNPT devices, which makes it difficult for healthcare providers to determine if ciNPT is beneficial to their practice specialty. This systematic review and meta-analysis assessed the impact of ciNPT on SSI occurrence after vascular surgery via groin incision. Furthermore, the impact of ciNPT use on SSI rates in patients at high risk or normal risk for surgical site complications and Szilagyi I–III infection classification^13^ following vascular surgery via groin incision was also assessed.

## Methods

The review conformed to the statement and reporting check list of the Preferred Reporting Items for Systematic Reviews and Meta-Analyses.^14^ The systemic literature review and meta-analysis were conducted using a previously unpublished internal protocol to assess the effect of ciNPT (PREVENA™ Incision Management System; KCI, San Antonio, TX) versus traditional postsurgical dressing use on SSI rates over closed groin incisions following vascular surgery. Secondary analyses assessed SSI rates in specific patient groups (high-risk vs. normal-risk) or Szilagyi (I, II, and III) outcomes. The high-risk and low-risk analyses evaluated the effect of ciNPT on SSI. “High-risk” subgroup analysis included studies that specifically recruited patients at a higher risk for wound infections. Inclusion criteria restricted the eligible patients to those with predetermined risk factors. The “Normal-risk” subgroup analysis included studies that did not restrict patient enrollment to only those with risk factors for wound infections but rather patients with any comorbidity profile were eligible.

Additional analyses were done to examine the effect of ciNPT on Szilagyi I, II, and III grade infections. Studies were included if they classified and reported the wound infections as Szilagyi I, II, and III grades. Grade I infections only involve the skin, grade II infections extend to the subcutaneous tissue without reaching the vessels, and grade III infections involve the artery.^10^

### Systematic literature search

A systematic literature search using PubMed, OVID, EMBASE, and QUOSA was performed on 3 January 2019. Literature between 1 January 2005 and 31 December 2018 was assessed. The following search terms were used: “negative pressure wound therapy” OR “vacuum-assisted closure” OR “negative pressure therapy” OR “NPWT”) AND (“Prevena” OR “ciNPT” OR “Prophylactic NPWT” OR “Preventive NPWT” OR “incision management” OR “incisional management” OR “closed incision negative pressure wound therapy” OR “closed incision negative pressure therapy.”

Inclusion criteria were abstracts or manuscripts written in English, published study, conference abstract, randomized controlled trial (RCT), comparison of ciNPT use over closed groin incisions to traditional postoperative dressings, endpoint/outcome of SSI, and a study population ≥10. Exclusion criteria included meta-analyses, preclinical studies (animal or bench studies), veterinary studies, pediatric patient population, study population <10, use of non-ciNPT device, comparative studies without randomization, and studies without traditional postoperative dressing control.

Studies were selected for inclusion following a review of the titles and abstracts to initially identify studies for further review. The full text of the articles was assessed for eligibility by two independent reviewers (LG and ME). When disagreement occurred, a third person (JR) reviewed the article, and a consensus on eligibility was decided.

Data extraction from all eligible studies was completed by one reviewer (LG) and was checked by a second independent reviewer (ME). Disagreements were resolved by the discussion between the two reviewers, or a third reviewer (JR) was brought in for review and discussion. Extracted data included: funding source, bias assessments, date range of the study, surgical procedures, number of patients/incisions enrolled, number of patients/incisions analyzed, high-risk patient enrollment characteristics, control type, treatment days, patient characteristics and comorbidities, differences in baseline characteristics, SSI measurement follow-up time, SSI definition/classification, number SSI, and type of SSI.

Each study included in the meta-analysis was assessed for bias in selection (randomization and allocation concealment), performance (blinding of participants and personnel, blinding of outcome assessments), attrition (loss to follow-up or incomplete outcome data), and reporting (comparison of reported results to endpoints defined in the protocol). The Cochrane Collaboration tool for assessing the risk of bias using the designation of low risk, high risk, or unclear was used.

### Statistical analysis

The meta-analyses were performed by calculating odds ratios (OR) using random effect models to assess the effect of ciNPT versus SOC on vascular groin incision SSIs. Subgroup analyses for patients designated as high-risk, normal-risk, and Szilagyi I, II, and III outcomes were also performed. Weighted odds ratios and 95% confidence intervals (CI) were calculated to pool study and control groups in each publication for analysis. The outcomes were measured using a binary variable. Treatment effects were combined using Mantel-Haenszel (M-H) odds ratios as the summary statistics, and a random effects model was used for each analysis performed. For each meta-analysis, the Chi-Square test of independence was used to assess heterogeneity. However, regardless of the heterogeneity assessment, the more conservative random effects model was used for each analysis for sensitivity analyses. All analyses were performed using RevMan Version 5.3 software (Copenhagen: The Nordic Cochrane Centre, The Cochrane Collaboration, 2014).

Funnel plots were used to assess the selection, identification, and publication bias by displaying the OR by the standard error of each study. If applicable, the funnel plots were generated for the subgroup analyses.

## Results

### Literature search results

**Figure 1.**
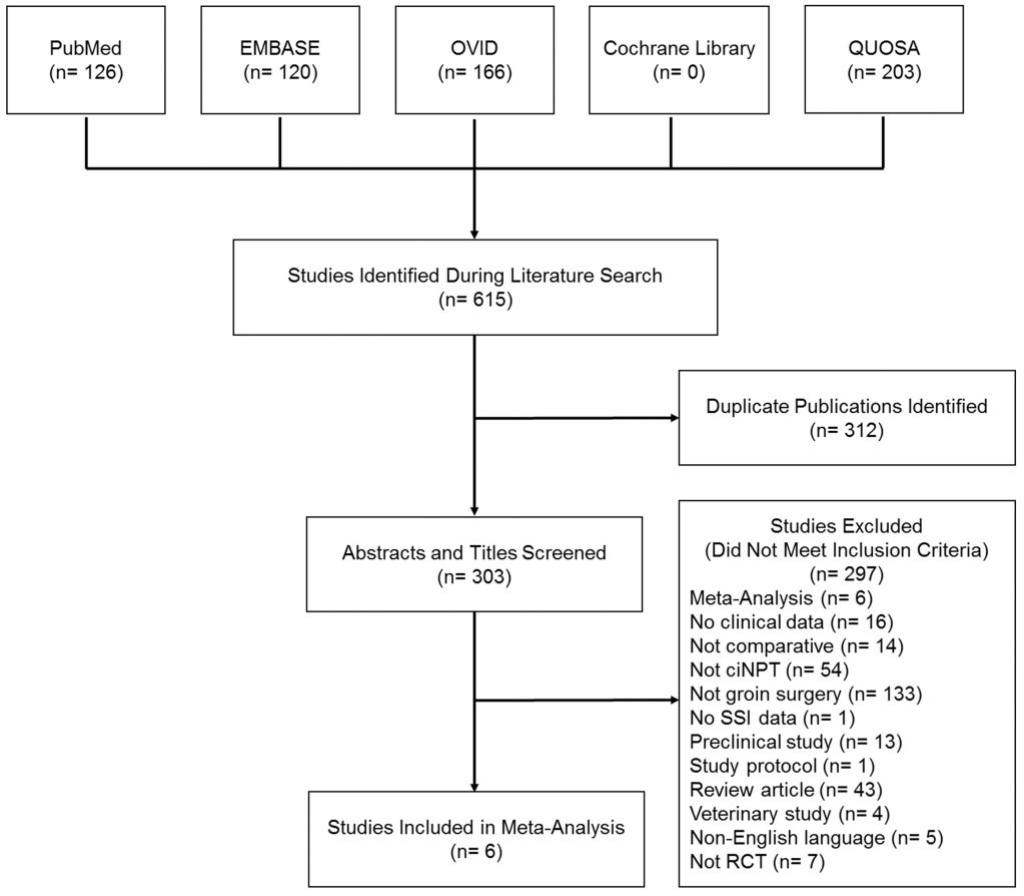
A PRISMA flowchart showing the process of identifying articles for inclusion in the systematic review and meta-analysis. ciNPT: closed incision negative pressure therapy; SSI: surgical site infection; RCT: randomized controlled trial.

A total of 615 publications were identified during the literature search. After removal of 312 duplicate publications, 303 abstracts and titles were screened against the inclusion and exclusion criteria. Reasons for exclusion are listed in Figure 1. Six RCTs were included in the analysis. The screening process is shown in Figure 1. There was a total of 733 incisions, of which 362 (49.4%) received ciNPT and 371 (50.6%) received standard of care.

### Description of studies

Characteristics of the six included studies are presented in Table 1. Five of the six studies reported use of longitudinal incision for the groin surgical incision. Incisions in the ciNPT treatment group received ciNPT for at least five days in all studies. Control treatments included absorbent dressings, sterile adhesive wound dressings, gauze, and conventional adhesive plaster. The frequency or duration of treatment in the control arms ranged from ≤ 2 days total to daily dressing changes. Of the six studies, two reported no device failures or adverse effects of the device.^11,15^ One study reported that one patient had the device removed early owing to an inability to achieve an adequate seal postoperatively.^16^ Three studies did not include information on device failure or adverse effects of the device.^3,17,18^

**Table 1.** Study characteristics of randomized controlled trials included in the meta-analysis.

Study	Vascular surgical procedure/type	Number of patients/incisions	Patient high-risk inclusion factors	Baseline population differences	Control treatment	ciNPT treatment days	Control treatment days
Engelhardt et al.^15^	Femoral cutdown	132	N/A	N/A	Absorbent dressing	5 days	≤2 days
Gombert et al.^11^	Vascular surgery for PAD	188	Tobacco use, HTN, CHD, MI, DM, dyslipidemia, hyperhomocysteinemia, CRF, reoperation	Higher patient age and higher rate of DM in ciNPT group	Sterile adhesive wound dressing	5–7 days	Changed daily
Kwon et al.^18^	Elective surgery, aortofemoral procedure, femoral-popliteal bypass	119	BMI > 30 kg/m^2^, pannus, fungal skin infection, reoperation, prosthetic vascular graft, poor nutrition, immunosuppression medication, poorly controlled DM (HbA1c > 8%)	N/A	Gauze covered by a transparent film dressing	5 days	2 days followed by dry gauze changed daily
Lee et al.^16^	Revascularization, femoral artery, bypass, endarterectomy, aorto-bifemoral bypass	102	BMI > 30 kg/m^2^, reoperation, ischemic tissue loss	ciNPT group had high rate of COPD and females	Sterile gauze dressing	First day of discharge or day 8	2 days followed by daily dressing changes
Pleger et al.^3^	Femoral artery EVAR/TEVAR, revascularization, bilateral procedures	129	Patients > 50 years, DM, renal insufficiency, malnutrition, obesity, COPD	ciNPT group had higher patient age and higher rate of CAD	Conventional adhesive plaster	5–7 days	Changed daily
Sabat et al.^17^	Open vascular surgery	63	N/A	N/A	Gauze and transparent film dressing	5 days	NR

ciNPT: closed incision negative pressure therapy; PAD: peripheral artery disease; EVAR: endovascular aneurysm repair; HTN: hypertension; CHD: coronary heart disease; MI: myocardial infarction; CRF: chronic renal failure; BMI: body mass index; NR: not reported; N/A: not applicable; DM: diabetes mellitus; COPD: chronic obstructive pulmonary disease; TEVAR: thoracic endovascular aortic repair; CAD: coronary artery disease.

Four of the six studies restricted inclusion to patients at a higher risk for developing SSIs.^3,11,16,18^ Study inclusion risk factors included tobacco use, hypertension, diabetes, obesity, immunosuppression, reoperation, renal insufficiency, malnutrition, and select other comorbidities. Two studies did not restrict patient enrollment to patients deemed at high risk for SSI development.^15,17^ Despite randomization, three studies reported significant differences in baseline characteristics. Higher rates of possible SSI risk factors were found in the ciNPT treatment arms.^3,11,16^

One RCT was multicenter,^11^ while the rest were performed at a single center.^3,15–18^ The Kwon et al. study was an interim analysis conducted after 80% of planned enrollment; the study met the prespecified stopping guideline and was stopped at the interim analysis prior to full enrollment.^18^ The Sabat et al. study was a published abstract reporting on results from the midpoint of RCT enrollment.^17^

Follow-up time for the primary outcome assessment of SSI ranged from 30 to 42 days in five studies. One study had a follow-up period of four months. All but one study reported the classification of SSIs with the Szilagyi Classification. In all studies included in the analysis, rates of SSIs were lower in the ciNPT group compared to the control groups (Table 2).

**Table 2.** Rates of surgical site infections per study.

Study	SSI assessment time	Definition/classification of SSI	Number analyzed of patients/incisions		Overall infection (%)	
			ciNPT	Control	ciNPT	Control
Engelhardt et al.^15^	42 days	Szilagyi classification	64	68	9 (14.0%)	19 (28.0%)
Gombert et al.^11^	30 days	Szilagyi classification	98	90	13 (13.2%)	30 (33.3%)
Kwon et al.^18^	30 days	Szilagyi classification	59	60	6 (10.1%)	12 (21.6%)
Lee et al.^16^	30 days	CDC definition/Szilagyi classification	53	49	6 (11.0%)	9 (18.0%)
Pleger et al.^3^	30 days	Szilagyi classification	58	71	5 (8.6%)	30 (42.3%)
Sabat et al.^17^	4 months	Not reported	30	33	2 (6.7%)	7 (21.2%)

ciNPT: closed incision negative pressure therapy; SSI: surgical site infection; CDC: Centers for Disease Control and Prevention.

### Risk of bias

**Figure 2.**
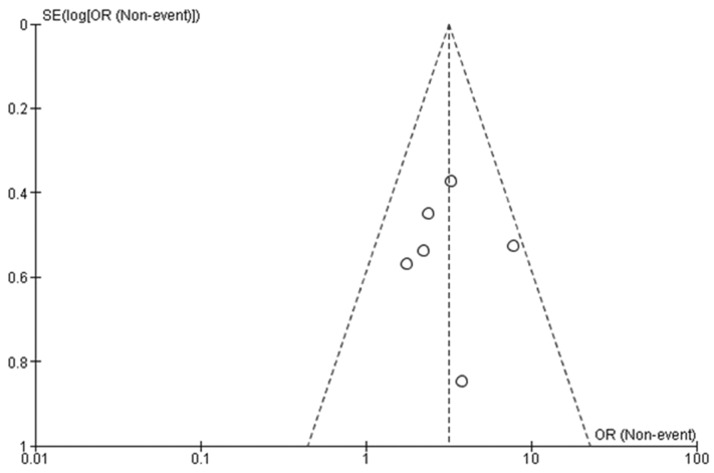
Funnel plot of studies included in the meta-analysis. Each circle indicates a single study. Dashed lines indicate 95% confidence interval. OR: odds ratio.

In four of the six studies, the authors adequately described the randomization methods and the allocation masking and were considered low risk of selection bias.^11,15,16,18^ All six studies had a high risk for bias due to the inability to blind participants and personnel to treatment due to the ciNPT device. Two studies were at high risk of outcomes assessment bias as the outcomes assessments were not blinded.^11,18^ Assessment bias was low in all five published manuscripts^3,11,15,16,18^ compared to the published abstract which did not provide sufficient information to correctly make an assessment.^17^ Three studies^11,16,18^ were low risk for selective reported, but it was unclear for the remaining three studies (Table 3).^3,15,17^ The funnel plot of the six studies included in the meta-analysis indicates that there is minimal risk of publication bias across the studies (Figure 2).

**Table 3.** Risk of bias within studies.

Study	Randomization method	Allocation masking	Blinding of participants and personnel	Blinded outcomes assessments	Assessment bias (enrolled vs. SSI assessment)	Assessment bias (results vs. defined endpoints)
Engelhardt et al.^15^	Low risk	Low risk	High risk	Unclear risk	Low risk	Unclear risk
Gombert et al.^11^	Low risk	Low risk	High risk	High risk	Low risk	Low risk
Kwon et al.^18^	Low risk	Low risk	High risk	High risk	Low risk	Low risk
Lee et al.^16^	Low risk	Low risk	High risk	Low risk	Low risk	Low risk
Pleger et al.^3^	Unclear risk	Unclear risk	High risk	Unclear risk	Low risk	Unclear risk
Sabat et al.^17^	Unclear risk	Unclear risk	High risk	Unclear risk	Unclear risk	Unclear risk

SSI: surgical site infection.

### Primary outcome

**Figure 3.**
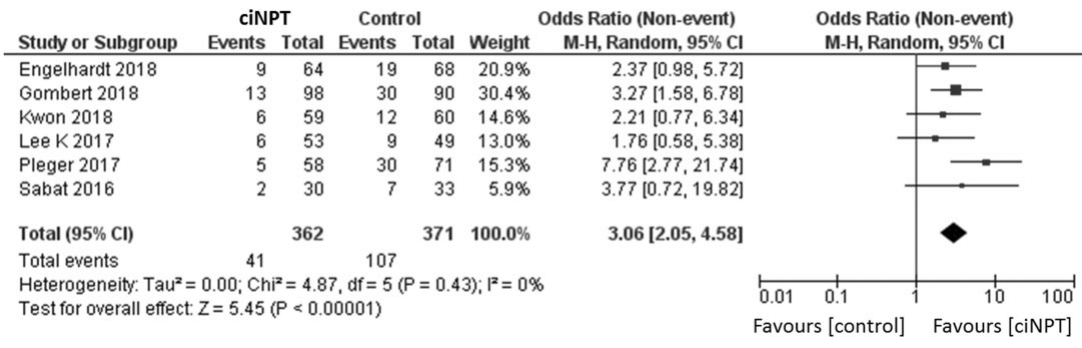
Surgical site infection forest plot comparing ciNPT and standard dressing use. Each study is displayed with the number of events and sample size. Total (95% CI) represented the summed sample sized, and the pooled OR calculated using a random effect model to adjust for the between-study heterogeneity. ciNPT: closed incision negative pressure therapy; CI: confidence interval.

**Figure 4.**
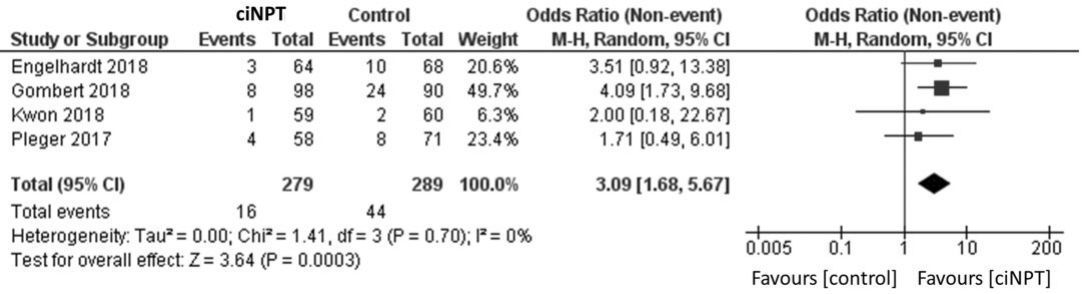
Surgical site infection forest plot comparing ciNPT and standard dressing use in Szilagyi I patients. Each study is displayed with the number of events and sample size. Total (95% CI) represented the summed sample sized, and the pooled OR calculated using a random effect model to adjust for the between-study heterogeneity. ciNPT: closed incision negative pressure therapy; CI: confidence interval.

**Figure 5.**
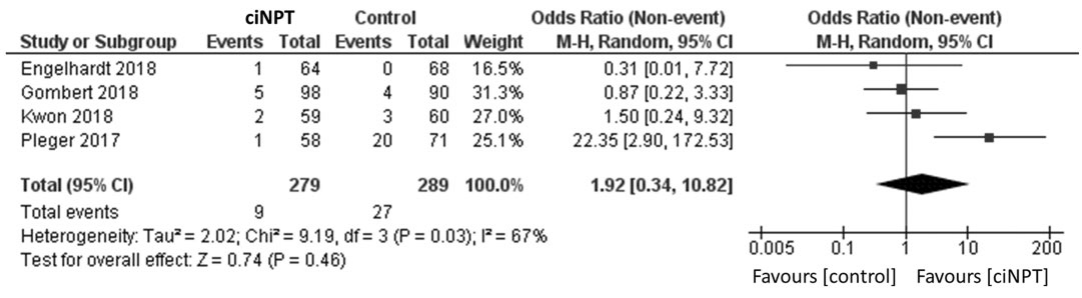
Surgical site infection forest plot comparing ciNPT and standard dressing use in Szilagyi II patients. Each study is displayed with the number of events and sample size. Total (95% CI) represented the summed sample sized, and the pooled OR calculated using a random effect model to adjust for the between-study heterogeneity. ciNPT: closed incision negative pressure therapy; CI: confidence interval.

**Figure 6.**
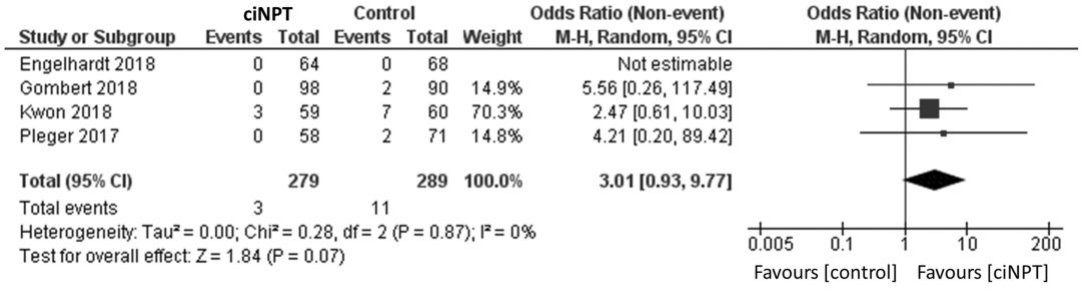
Surgical site infection forest plot comparing ciNPT and standard dressing use in Szilagyi III patients. Each study is displayed with the number of events and sample size. Total (95% CI) represented the summed sample sized, and the pooled OR calculated using a random effect model to adjust for the between-study heterogeneity. ciNPT: closed incision negative pressure therapy; CI: confidence interval.

Using a random effects model, patients treated with ciNPT had a lower risk of developing an SSI when compared to the control arm (OR = 3.06, 95% CI [2.05, 4.58], *p* < 0.05; Figure 3 and Table 4). Four studies reported data by the Szilagyi classification for SSIs.^3,11,15,18^ For grade I infections, a lower risk of infection was found for patients treated with ciNPT (OR = 3.09, 95% CI [1.68, 5.67], *p* < 0.05; Figure 4). No significant differences were found in the risk of Szilagyi Grade II infections (OR = 1.92, 95% CI [0.34, 10.82], *p* = 0.46; Figure 5) or Szilagyi Grade III infections (OR = 3.01, 95% CI [0.93, 9.77], *p* = 0.07; Figure 6).

**Table 4.** Overview of meta-analyses results.

Outcome or subgroup	Studies	Subjects/incisions	Effect estimate OR (95% CI)	*p*	*I* ^2^
Overall	6	733	3.06 (2.05, 4.58)	**<0.00001**	0%
High risk	4	538	3.22 (1.79, 5.78)	**<0.0001**	32%
Normal risk	2	195	2.62 (1.20, 5.72)	**0.02**	0%
Szilagyi I	4	568	3.09 (1.68, 5.67)	**0.0003**	0%
Szilagyi II	4	568	1.92 (0.34, 10.82)	0.46	67%
Szilagyi III	4^a^	568	3.01 (0.93, 9.77)	0.07	0%

*I*^2^ = measure of heterogeneity. Bold p values indicate statistically significant *p* value.

^a^One study in the Szilagyi III analysis reported no events in either the ciNPT or control group.

### Subgroup analysis

**Figure 7.**
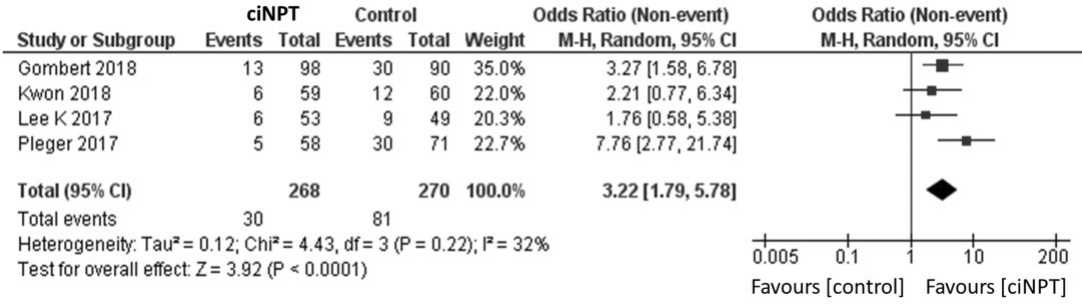
Surgical site infection forest plot comparing ciNPT and standard dressing use in patients at high-risk for postsurgical infection development. Each study is displayed with the number of events and sample size. Total (95% CI) represented the summed sample sized, and the pooled OR calculated using a random effect model to adjust for the between-study heterogeneity.

**Figure 8.**
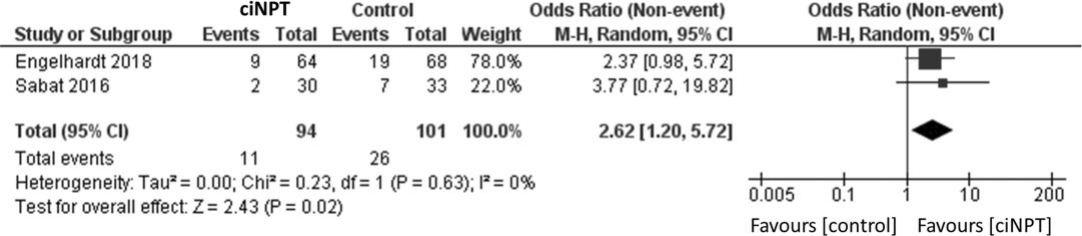
Surgical site infection forest plots comparing ciNPT and standard dressing use in patients with normal risk for postsurgical infection development. Each study is displayed with the number of events and sample size. Total (95% CI) represented the summed sample sized, and the pooled OR calculated using a random effect model to adjust for the between-study heterogeneity. ciNPT: closed incision negative pressure therapy; CI: confidence interval.

The analysis of the four studies restricting enrollment to patients at a higher risk of SSI found a significantly lower risk of infection in patients treated with ciNPT (OR = 3.22, 95% CI [1.79, 5.78], *p* < 0.05; Figure 7).^3,11,16,18^ The findings were similar for the analysis of the two studies that did not restrict enrollment (OR = 2.62, 95% CI [1.20, 5.72], *p* < 0.05; Figure 8).^15,17^

## Discussion

SSI after groin incision is common and creates additional complications for patients (such as increased risk of limb loss and patient mortality) in addition to being a financial burden on healthcare systems. Use of ciNPT has been utilized with positive clinical benefit in a variety of surgical incision types.^6–12^ This meta-analysis assessed the impact of a single ciNPT device use on SSI rates in vascular surgery with groin incision compared to traditional postsurgical dressings in RCTs.

The systematic review identified six RCTs for analysis. The study populations examined displayed a variety of comorbidities and were representative of the typical patient populations undergoing vascular surgery with groin incision. As such, the statistically significant reduction of SSI rates following ciNPT use may be expected in similar patient populations.

Previous literature has recommended ciNPT use in patients at high risk of developing SSIs.^19^ These patients typically have multiple comorbidities, general incision-related risk factors, and procedure-related risk factors. Indeed, the meta-analysis focused on patients deemed at high risk for SSI development showed a significant reduction in SSI rates following ciNPT use compared to traditional postoperative dressings. Interestingly, the results also indicated that patients with a normal risk for SSI development showed fewer infections with ciNPT compared to traditional postoperative dressings.

Our meta-analysis also examined the effect of ciNPT use on Szilagyi I, II, and III SSI rates compared to traditional postoperative dressings. Four out of six RCTs reported data on Szilagyi graded SSIs. Overall, a significantly reduced rate of Szilagyi I graded SSIs were reported in the ciNPT groups compared to traditional postoperative dressings. However, the comparison of grade II and III SSI rates did not show a statistically significant decreased rate in the ciNPT group, most likely based on the low number of patients assessable in each study suffering from these severe SSIs. Each of the RCTs included in the analysis were designed to look at the occurrence of all SSIs as the primary outcome and were therefore sized accordingly. The studies were not sufficiently powered to evaluate differences for the separate Szilagyi grades, specifically grades II and III which occur at much lower rates.

Recently, a meta-analysis examining the use of negative pressure therapy over vascular incisions has been published.^20^ While our analysis focused on one specific device, the published study included therapy units from two different manufacturers, potentially leading to a lack of homogeneity in the interventional group. Although the same tools have been used to assess the risk of bias, different risk bias findings have been reported. Unfortunately, based on the available information and data, the reason for these differences was not determinable, especially the risk of bias for the study of Gombert et al.^11^ While assessing the original raw data of the Gombert study, the high risk of bias as reported by Svensson-Björk et al.^20^ could not be confirmed. Despite the discrepancies, the Svensson-Björk meta-analysis indicated that use of incision negative pressure wound therapy after groin incision in vascular surgery reduced the incidence of SSI compared to traditional postoperative dressings.^20^ This finding is similar to our own.

Up to now, the estimated and desired beneficial use of ciPNT regarding a shorter length of stay, reduced SSIs, and costs could not be confirmed. Kwon et al. suggest a positive impact of ciNPT in high-risk patients and reduced costs during the first 30 days after surgery.^18^ No further high-quality evidence specific for vascular surgery is available regarding a potential economic advantage. Furthermore, the transferability of knowledge regarding reduced treatment costs in different surgical settings is limited.

## Limitations

One study limitation is the inclusion/exclusion criteria differences between the six RCTs utilized for the meta-analysis. However, heterogeneity of the data was assessed for each meta-analysis performed. The heterogeneity was 0% for the overall, normal-risk, Szilagyi I, and Szilagyi III analyses indicating similar study populations across these RCTs. Heterogeneity was 32% for the high-risk analysis and 67% for the Szilagyi II analysis, implying a more diverse patient population. To minimize any potential population heterogeneity, all meta-analyses were performed using conservative random effects models to help account for potential variations across the study populations.

Similarly, there is always a risk for selection bias in meta-analysis articles. However, the authors followed a well-defined systematic literature search protocol to help minimize potential bias. A funnel plot of the six studies included in the meta-analysis indicated that there is minimal risk of publication bias.

Another potential limitation for our study was the blinding of the assessing healthcare provider. Wound assessment was performed by subjective appraisal. The nature of ciNPT meant that double-blinded treatment was not possible. However, measures were taken to minimize potential bias at least in one RCT.

Regarding the grading of SSI, the implantation of two or more scoring systems would have been favorable. The Szilagyi classification could be supported by ASEPSIS criteria, which would have enabled a less subjective and more reliable, structural assessment of the wound conditions.^21^ As the Szilagyi classification is well known, clinically established, and present in the major part of studies dealing with SSI, this point remains debatable.

Unfortunately, this meta-analysis could not assess potential safety concerns (such as dehiscence or reoperations) with the utilization of ciNPT, as only three of the studies^3,16,18^ included in the analysis reported on adverse events. However, this may be due to ciNPT having a similar safety profile as traditional postoperative dressings.

## Conclusion

For these meta-analyses, ciNPT usage demonstrated a statistically significant reduction in the incidence of SSI relative to traditional postsurgical dressings in patients undergoing vascular surgery with groin incisions. Future studies further assessing cost-effectiveness and adverse events following ciNPT use compared with traditional postsurgical dressings are required.
